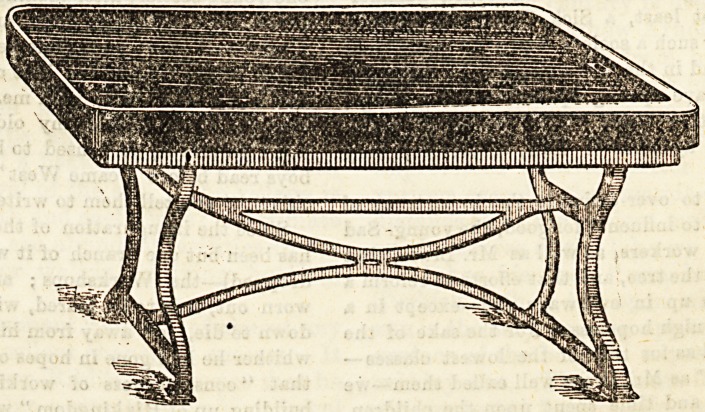# A New Post-Mortem Table

**Published:** 1892-04-23

**Authors:** 


### FURNITURE AND FITTINGS.

### A NEW POST-MORTEM TABLE.

To say that the most scrupulous cleanliness in every detail is a matter of the first importance in a room set apart for post-mortem examinations appears to be a somewhat trite truism. But, nevertheless, when we come to examine the interiors of such rooms, in the majority of hospitals, we find that the construction and fittings are generally of such a nature as to favour rather than prevent the wholesale storage of dirt, and to make the task of efficient cleaning difficult, if not impossible. The dangers involved in the work of autopsy are very great, even when the greatest care is exercised by those engaged in it; and everything therefore that can be done to lessen the possibilities of harm to the living should be done.

To this end everything in a post-mortem room should be made of a smooth, washable, and non-absorbent surface as possible. Walls of glazed brick, with hard cement joints, floor of asphalte or marble terazzo work, window frames of iron, and doors, which must be of wood, either of oak, or if of deal, well painted and varnished. There should be no recesses for pipes, which should all be fixed free of the walls, so that the surface of the wall behind the pipes as well as every part of the latter can be freely got at in cleaning. The drainage should be conducted in open channels sunk in the floor to the outside, and the floor should slope from the walls to the channels so that it can be freely swilled down with a hose pipe.

Post-mortem tables are commonly made in this country of slate supported on wooden framework. The disadvantage of slate as a material for this purpose is that it is not absolutely non-absorbent, and that it will not take a polish. It is also liable to split when in sudden contact with hot water. Abroad the custom is far more general to use marble for this purpose, the supports, however, being usually made of wood.

The post-mortem tables at the Middlesex Hospital were devised with a view to obtain a perfectly washable, impervious structure in every part. The slab itself is of Belgian fossil marble, a dense hard limestone, capable of taking a high polish and of standing rapid and extreme variations of temperature. This slab is slightly raised in the centre and slopes to a channel at each side, the channels again falling to one end and meeting at an outlet, which is furnished with a specially-made grating curved to the contour of the channel. Through this grating the water passes down a copper pipe into the open floor channel below. The supports are made of cast-iron so designed that every portion is rounded, and there are no angles or ledges anywhere. Over each table, dependent from the ceiling hangs an india-rubber tube with a brass rose; both hot and cold water are laid on to this tube from a mixing valve on the wall, so that either hot or cold, or both together, to any required temperature, can be used. Around the sides and ends of the tables are india-rubber mats in place of the ordinary wooden trellis standing boards.

The shelves which run around the walls upon which much pathological work is done are also of the same kind of marble, and are supported on specially-made iron brackets.

The other fittings necessary for a post-mortem room are lavatory basins, a sink, and an instrument case. At the Middlesex Hospital the lavatory basins are of white porcelain with a marble top; the basins and the marble are supported on iron brackets, and the space underneath is entirely open. The sink is of fire-clay, glazed inside and outside, and like the lavatory basins is supported on brackets and has no enclosure underneath. The instrument cupboard is of mahogany, with air-tight glazed doors, and has a sloping top.

For washing down the floor and walls, a small hydrant with a hose-pipe is provided, and for flushing out the floor channels two small flushing cisterns are fixed on the walls, one at each end of the room.

## Figures and Tables

**Figure f1:**